# Dietary patterns and the risk of CVD and all-cause mortality in older British men

**DOI:** 10.1017/S0007114516003147

**Published:** 2016-09-13

**Authors:** Janice L. Atkins, Peter H. Whincup, Richard W. Morris, Lucy T. Lennon, Olia Papacosta, S. Goya Wannamethee

**Affiliations:** 1 Department of Primary Care and Population Health, University College London, London NW3 2PF, UK; 2 Epidemiology and Public Health Group, Medical School, University of Exeter, RILD Building, Barrack Road, ExeterEX2 5DW, UK; 3 Population Health Research Centre, Division of Population Health Sciences and Education, St George’s, University of London, London SW17 0RE, UK; 4 School of Social and Community Medicine, University of Bristol, Bristol BS8 2PS, UK

**Keywords:** *A posteriori* dietary patterns, CVD, Mortality, Older adults, Principal component analysis

## Abstract

Dietary patterns are a major risk factor for cardiovascular morbidity and mortality; however, few studies have examined this relationship in older adults. We examined prospective associations between dietary patterns and the risk of CVD and all-cause mortality in 3226 older British men, aged 60–79 years and free from CVD at baseline, from the British Regional Heart Study. Baseline FFQ data were used to generate thirty-four food groups. Principal component analysis identified dietary patterns that were categorised into quartiles, with higher quartiles representing higher adherence to the dietary pattern. Cox proportional hazards examined associations between dietary patterns and risk of all-cause mortality and cardiovascular outcomes. We identified three interpretable dietary patterns: ‘high fat/low fibre’ (high in red meat, meat products, white bread, fried potato, eggs), ‘prudent’ (high in poultry, fish, fruits, vegetables, legumes, pasta, rice, wholemeal bread, eggs, olive oil) and ‘high sugar’ (high in biscuits, puddings, chocolates, sweets, sweet spreads, breakfast cereals). During 11 years of follow-up, 899 deaths, 316 CVD-related deaths, 569 CVD events and 301 CHD events occurred. The ‘high-fat/low-fibre’ dietary pattern was associated with an increased risk of all-cause mortality only, after adjustment for confounders (highest *v.* lowest quartile; hazard ratio 1·44; 95 % CI 1·13, 1·84). Adherence to a ‘high-sugar’ diet was associated with a borderline significant trend for an increased risk of CVD and CHD events. The ‘prudent’ diet did not show a significant trend with cardiovascular outcomes or mortality. Avoiding ‘high-fat/low-fibre’ and ‘high-sugar’ dietary components may reduce the risk of cardiovascular events and all-cause mortality in older adults.

Diet is a well-established major modifiable risk factor for cardiovascular morbidity and mortality and may be particularly important in older adults, who are at higher risk of chronic disease^(^ ^1^ ^–^ ^4^ ^)^. Historically, studies investigating the associations between diet and risk of CVD or mortality have focused on single food items or specific dietary nutrients^(^ ^3^ ^–^ ^5^ ^)^; however, more recently, research has tended to focus on overall dietary patterns to reflect the complex multidimensional nature of diets consumed in the population and to examine the combined effects of the consumption of various foods/nutrients^(^ ^6^ ^–^ ^8^ ^)^. The following two main approaches have been developed to assess dietary patterns: (1) *a priori* approaches, which are hypothesis oriented or theoretically defined, as they use available scientific evidence to generate predefined dietary scores or indices based on dietary recommendations or guidelines; and (2) *a posteriori* approaches, which are data driven or exploratory, as dietary patterns are derived from the available data based on methods such as principal component analysis or cluster analysis^(^ ^7^ ^,^ ^9^ ^)^. However, few studies have examined the relationships between dietary patterns and morbidity or mortality in older adults specifically^(^ ^10^ ^,^ ^11^ ^)^.

We have previously applied *a priori* diet quality scores to the British Regional Heart Study (BRHS), showing that older men with higher adherence to the Elderly Dietary Index (a modified Mediterranean diet score) were at lower risk of CVD and all-cause mortality^(^ ^12^ ^)^. However, compared with *a priori* dietary patterns, *a posteriori* methods of defining dietary patterns have the advantage of not making any previous assumptions or hypotheses but use the existing data to characterise total diet, so that patterns describe the eating behaviour of a population^(^ ^9^ ^)^. Principal component analysis is one common method of deriving *a posteriori* dietary patterns and is a data-reduction technique, which identifies foods that are frequently consumed together and aggregates food items or groups on the basis of the degree of correlation with one another^(^ ^7^ ^,^ ^9^ ^)^. It has been suggested that principal component analysis may generate more meaningful and interpretable dietary patterns than cluster analysis (an alternative *a posteriori* method that separates individuals into mutually exclusive groups based on the differences in dietary intake) as it has higher statistical power and is less influenced by extreme values^(^ ^8^ ^,^ ^13^ ^)^.

Defining dietary patterns *a posteriori* has typically identified two major types of patterns – healthy (‘prudent’) and unhealthy (‘Western’) diets^(^ ^13^ ^–^ ^15^ ^)^. Healthy/prudent dietary patterns have tended to show inverse associations with CVD and mortality risk^(^ ^13^ ^–^ ^15^ ^)^, whereas unhealthy/Western patterns have either shown positive associations or no significant association at all^(^ ^13^ ^–^ ^16^ ^)^. Although the relationship between *a posteriori-*defined dietary patterns and the risk of CVD and mortality has been examined in middle-aged populations, few studies have been carried out in older adults in particular^(^ ^17^ ^)^, with a particular paucity of studies in older British populations^(^ ^16^ ^)^. The aim of this study was therefore to examine the prospective associations between *a posteriori* dietary patterns, defined using principal component analysis, and the risk of CVD and mortality in a cohort of older British men.

## Methods

### Subjects and methods of data collection

The BRHS is a prospective study of CVD, in a socio-economically and geographically representative sample of 7735 British men, selected from twenty-four towns across Great Britain^(^ ^18^ ^,^ ^19^ ^)^. The cohort was initially examined in 1978–1980 and is predominantly comprised of white European ethnic origin (>99 %). This study used data from the 20-year re-examination of BRHS participants in 1998–2000, aged 60–79 years^(^ ^20^ ^)^. In total, 4252 men (77 % of survivors) completed a questionnaire answering questions on their lifestyle and medical history, completed a FFQ, attended a physical examination and provided a fasting blood sample. Of the 4252 men attending the physical examination, 723 men with prevalent heart failure, myocardial infarction (MI) or stroke at baseline were excluded (on the basis of previous diagnosis, according to self-report), leaving 3529 participants for inclusion in this study. Participants were followed-up for morbidity and mortality until 2010. All participants provided written informed consent, in accordance with the Declaration of Helsinki, and ethics approval was also obtained from relevant local research ethics committees.

### Dietary assessment

Baseline dietary intake was measured during 1998–2000 with a self-administered postal FFQ, which was developed for use in the World Health Organization’s Monitoring Trends and Determinants in Cardiovascular Disease Survey^(^ ^21^ ^)^. The FFQ has been validated against weighed food intake in British populations, and statistically significant correlation coefficients were observed between methods for all main nutrients of between 0·27 (total carbohydrate) and 0·75 (alcohol)^(^ ^22^ ^,^ ^23^ ^)^. Participants reported that they usually consumed eighty-six different food and drink items per week, which were reported in nine categories: 1, 2, 3, 4, 5, 6 or 7 d/week, monthly or rarely/never. A validated computer programme was used to calculate the total macronutrient and micronutrient intakes of all foods reported as consumed in the FFQ, and hence the total energy intake^(^ ^24^ ^)^. This computer programme multiplied food frequency by standard portion sizes for each food and by the nutrient composition of the food obtained from the UK food composition tables^(^ ^25^ ^)^. The distribution of total energy intakes was checked for any extreme values. However, all were within a range compatible with a normal lifestyle (2092–33 472 kJ (500–8000 kcal)/d in men^(^ ^26^ ^)^), and therefore no exclusions were made on this basis.

The eighty-six food items in the FFQ were aggregated into thirty-four mutually exclusive food groups on the basis of the similarity of food types and nutrient composition; these were comparable with food groups used previously for a nationally representative dietary survey of British adults^(^ ^27^ ^)^. Individual food items were summed to generate a total score for each of the thirty-four food groups. The food groups were generated if at least one of the food items within the group was not missing. A list of these thirty-four food groups generated from the FFQ, together with their units of measurement and range, is shown in the online Supplementary Table S1. Of the 3529 participants who attended the 20-year re-examination and were free from prevalent heart failure, MI or stroke, 303 participants with missing data on any of the thirty-four food groups were excluded. However, the 303 participants with missing food group data were not significantly different from the 3226 included participants with respect to diet quality (age-adjusted OR 0·82; 95 % CI 0·56, 1·22, *P*=0·34; for difference in the proportion consuming fruits and vegetables daily) or CVD event risk (age-adjusted hazard ratio (HR) 1·00; 95 % CI 0·77, 1·31, *P*=0·98).

### Principal component analysis

Principal component analysis was conducted using orthogonal varimax rotation on the thirty-four food groups generated from the FFQ in order to identify dietary patterns. Principal component analysis was performed in Stata 13.1 (StataCorp LP), using a correlation matrix that transformed the input variables (food groups) to *z*-scores^(^ ^28^ ^)^ to account for the different scales of measurement of the food groups used. In all, three principal components were retained on the basis of having an eigenvalue>1, the scree plot of eigenvalues and the interpretability of the rotated factors^(^ ^29^ ^)^. Food groups with factor loading of more than 0·20 or less than −0·20 were considered to be important contributors to the dietary pattern, based on cut-off values used in previous studies^(^ ^30^ ^)^. The factor scores for each dietary pattern were calculated for each participant by summing the intakes of the food groups weighted by their factor loadings. The higher the score, the closer the diet to the dietary pattern, and the lower the score the further the diet from the dietary pattern. Participants were then classified into quartiles of adherence to each of the three major dietary patterns, with higher quartiles representing higher adherence to the dietary pattern.

### Covariates

Measures of cigarette smoking, physical activity and alcohol intake were self-reported via a questionnaire at the 20-year examination in 1998–2000, as described^(^ ^31^ ^–^ ^33^ ^)^. Participants were classified into four cigarette smoking groups (never smoked; long-term ex-smokers, >15 years; recent ex-smokers, ≤15 years; current smokers)^(^ ^31^ ^)^. Current physical activity was classified into six groups on the basis of intensity and frequency of exercise (inactive; occasional; light; moderate; moderately vigorous; vigorous)^(^ ^32^ ^)^. Alcohol intake was classified into five groups on the basis of the number and frequency of alcoholic drinks consumed per week (none; occasional; light; moderate; heavy)^(^ ^33^ ^)^. Social class was measured using the baseline questionnaire in 1978–1980, based on the longest held occupation coded using the Registrar General’s occupational classification^(^ ^34^ ^)^. Participants were classified as manual, non-manual or armed forces. Systolic blood pressure (SBP), height and weight were assessed at the physical examination in 1998–2000. BMI was calculated, and participants were classified into four categories using WHO’s cut-off points (underweight, <18·5 kg/m^2^; normal weight, 18·5–24·99 kg/m^2^; overweight, 25–29·99 kg/m^2^; obese, ≥30 kg/m^2^)^(^ ^35^ ^)^. Blood samples were collected at the 20-year re-examination in 1998–2000, which allowed measurement of plasma concentrations of HDL and glucose, and two emerging cardiovascular risk factors: C-reactive protein (CRP), a marker of inflammation, was assayed by ultrasensitive nephelometry and von Willebrand factor (vWF), a marker of endothelial dysfunction, was measured with ELISA^(^ ^32^ ^)^. In addition, at the 20-year examination, participants were classified as having prevalent diabetes if they had a previous diagnosis, according to self-report. Complete case analysis was used to deal with any missing covariates.

### Follow-up and outcome ascertainment

Participants were prospectively followed-up for cardiovascular mortality and morbidity from re-examination in 1998–2000 to June 2010, and follow-up was achieved for 98 % of the cohort^(^ ^36^ ^)^. Information on deaths was collected through the National Health Service Central Register (death certificates coded using International Classification of Diseases, ninth revision (ICD-9)). Evidence regarding non-fatal events was obtained using ongoing reports from general practitioners and using biennial reviews of the patients’ medical records^(^ ^20^ ^)^. The four outcomes examined in this study were as follows: CHD events (diagnosis of fatal or non-fatal MI (ICD-9 codes 410-414)); CVD events (diagnosis of non-fatal MI (ICD-9 codes 410-414), non-fatal stroke (ICD-9 codes 430-438) or fatal CVD (ICD-9 codes 390-459)); CVD mortality (ICD-9 codes 390-459); and all-cause mortality. Participants were censored at date of death or at the end of the study period (June 2010).

### Statistical analysis

Baseline characteristics of the participants are presented by quartiles of the distribution of the three dietary patterns. The distribution of CRP was highly skewed and was log transformed. Cox proportional hazards regression models were fitted to assess the association between quartiles of adherence to dietary patterns and the risk of all-cause mortality, CVD mortality, CVD events and CHD events. Tests for trend of risk of outcomes across quartiles of dietary patterns were performed. All Cox models were tested for the proportional hazards assumption on the basis of Schoenfeld residuals, which was not found to be violated. Multivariable models were adjusted for potential confounders in a sequential manner, including age (model 1); energy intake, smoking status, alcohol intake, physical activity, social class and BMI (model 2); HDL, SBP and diabetes (model 3); and CRP and vWF (model 4). Age, energy intake, HDL-cholesterol, SBP, CRP and vWF were fitted as continuous variables. Smoking status, alcohol intake, physical activity, social class, BMI and diabetes were fitted as categorical variables. All statistical analyses were performed in Stata 13.1 (StataCorp LP).

## Results

The analyses were based on 3226 men, aged 60–79 years, who attended the 20-year re-examination, were free from prevalent heart failure, MI and stroke at baseline, and provided information on the thirty-four food groups. A total of three interpretable *a posteriori* dietary patterns identified by principal component analysis explained 20·8 % of the total variance in diet. The online Supplementary Table S2 shows the food group factor loadings for each of the three dietary patterns. The dietary pattern defined by the first principal component was labelled ‘high fat/low fibre’, and explained 7·9 % of the total variance. This dietary pattern was characterised by a high consumption of red meat, meat products, fried potato, white bread, eggs and beer (positive scoring coefficients) and a low intake of wholemeal bread (negative scoring coefficients). The second principal component reflected a ‘prudent’ diet, explaining 7·1 % of the variance, and was characterised by a high consumption of poultry, fish, vegetables, legumes, fruits, pasta and rice, wholemeal bread, eggs, sauces, soups and olive oil (positive scoring coefficients). The third principal component reflected a ‘high-sugar’ diet, and explained 5·8 % of the total variance. This dietary pattern was characterised by a high consumption of breakfast cereals, full-fat cheese, biscuits, puddings, chocolates, sweets and sweet spreads (positive scoring coefficients) and a low consumption of beer (negative scoring coefficients).

Adherence to the ‘high-fat/low-fibre’ dietary pattern showed a strong positive association with current smoking, heavy drinking, physical inactivity, manual social class, obesity, total energy intake, CRP and vWF and an inverse association with HDL. The prevalence of diabetes was also inversely associated with the ‘high-fat/low-fibre’ dietary pattern, which was an unexpected finding and may be an example of dietary change, secondary to the development of illness (Table 1). Adherence to the ‘prudent’ pattern was strongly, inversely associated with age, current smoking, physical inactivity, manual social class, CRP and vWF, and positively associated with HDL and diabetes (Table 2).

**Table 1:** Baseline characteristics of the British Regional Heart Study Participants by quartiles (Q) of a ‘high-fat/low-fibre’ dietary pattern, in 1998–2000 (Mean values and standard deviations; geometric mean and interquartile range (IQR))

	‘High-fat/low-fibre’ dietary pattern								
	Q1		Q2		Q3		Q4		
	Mean	sd	Mean	sd	Mean	sd	Mean	sd	*P*_trend_
*n*	807		806		807		806		
Age (years)	68·0	5·4	68·5	5·3	68·4	5·5	68·1	5·4	0·81
Energy intake (kJ)	7789·8	1612·1	8387·7	1758·1	9101·0	1864·0	10 662·5	2474·0	<0·001
Energy intake (kcal)	1861·8	385·3	2004·7	420·2	2175·2	445·5	2548·4	591·3	<0·001
Current smokers (%)	4·3		7·6		13·7		24·7		<0·001
Heavy drinkers (%)	1·3		1·3		3·0		5·8		<0·001
Physically inactive (%)	7·9		9·6		8·2		11·9		0·02
Manual social class (%)	32·5		41·8		53·4		69·4		<0·001
Obese (BMI≥30 kg/m^2^) (%)	14·5		15·4		15·9		18·8		0·02
SBP (mmHg)	149·9	24·5	149·5	23·7	150·7	23·0	150·5	24·3	0·40
HDL (mmol/l)	1·4	0·4	1·3	0·3	1·3	0·3	1·3	0·4	0·02
Glucose (mmol/l)	6·0	1·9	5·9	1·6	6·0	1·9	6·0	1·8	0·98
Diabetes (%)	8·2		6·9		5·2		5·4		0·01
CRP (mg/l)*									<0·001
Mean	1·3		1·5		1·9		2·0		
IQR	0·6–2·7		0·7–2·7		0·9–3·8		1·0–4·0		
vWF (IU/dl)	132·7	45·2	135·5	44·0	139·2	44·8	139·3	46·3	0·001

SBP, systolic blood pressure; CRP, C-reactive protein; vWF, von Willebrand factor.

* Log transformed.

**Table 2:** Baseline characteristics of the British Regional Heart Study Participants by quartiles (Q) of a ‘prudent’ dietary pattern, in 1998–2000 (Mean values and standard deviations; geometric mean and interquartile range (IQR))

	‘Prudent’ dietary pattern								
	Q1		Q2		Q3		Q4		
	Mean	sd	Mean	sd	Mean	sd	Mean	sd	*P*_trend_
*n*	807		806		807		806		
Age (years)	68·8	5·4	68·4	5·5	67·9	5·2	68·0	5·5	0·001
Energy intake (kJ)	8135·4	1931·8	8921·5	2074·8	9099·8	2153·9	9772·6	2420·4	<0·001
Energy intake (kcal)	1944·4	461·7	2132·3	495·9	2174·9	514·8	2335·7	578·5	<0·001
Current smokers (%)	21·4		13·0		9·5		6·4		<0·001
Heavy drinkers (%)	3·0		3·0		1·8		3·5		0·85
Physically inactive (%)	14·4		9·5		7·0		6·8		<0·001
Manual social class (%)	62·3		52·7		46·7		35·4		<0·001
Obesity (BMI≥30 kg/m^2^) (%)	15·5		16·5		16·9		15·8		0·82
SBP (mmHg)	149·7	23·9	150·5	23·9	149·4	23·8	150·9	23·9	0·50
HDL (mmol/l)	1·3	0·3	1·3	0·3	1·3	0·3	1·4	0·3	<0·001
Glucose (mmol/l)	5·9	1·9	6·0	1·7	6·0	1·7	6·1	2·0	0·20
Diabetes (%)	5·2		5·0		7·7		7·8		0·007
CRP (mg/l)*									<0·001
Mean	2·0		1·7		1·5		1·4		
IQR	0·9–4·0		0·8–3·6		0·7–3·0		0·7–2·6		
vWF (IU/dl)	140·7	45·8	140·2	46·7	134·7	43·3	131·0	44·0	<0·001

SBP, systolic blood pressure; CRP, C-reactive protein; vWF, von Willebrand factor.

* Log transformed.

Contrary to expectations, in unadjusted analyses, several behavioural variables (current smoking, heavy drinking and physical inactivity) and obesity were inversely associated with adherence to a ‘high-sugar’ dietary pattern (Table 3). To test whether the observed inverse association between manual social class and a ‘high-sugar’ diet could explain this, analyses were stratified into manual and non-manual occupational social class, but the inverse associations between a ‘high-sugar’ diet and adverse behavioural risk factors and obesity were still apparent within both groups (data not presented). Men in the highest quartile of the ‘high-sugar’ diet also had the lowest proportion of obese individuals, and it is therefore possible that obese men had made changes to their diet to reduce sugar intake. However, when analyses were stratified into obese and non-obese participants, inverse associations between a ‘high-sugar’ diet and adverse behavioural risk factors remained in both groups. Adherence to the ‘high-sugar’ diet was also significantly associated with age and intake of total energy and inversely associated with prevalent diabetes, glucose, HDL and CRP, but not significantly associated with SBP or vWF.

**Table 3:** Baseline characteristics of the British Regional Heart Study Participants by quartiles (Q) of a ‘high-sugar’ dietary pattern, in 1998–2000 (Mean values and standard deviations; geometric mean and interquartile range (IQR))

	‘High-sugar’ dietary pattern								
	Q1		Q2		Q3		Q4		
	Mean	sd	Mean	sd	Mean	sd	Mean	sd	*P*_trend_
*n*	807		806		807		806		
Age (years)	67·1	5·1	68·0	5·2	68·6	5·4	69·3	5·7	<0·001
Energy intake (kJ)	7762·6	1932·2	8347·5	1816·7	9205·6	1909·6	10606·9	2151·8	<0·001
Energy intake (kcal)	1855·3	461·8	1995·1	434·2	2200·2	456·4	2535·1	514·3	<0·001
Current smokers (%)	17·6		12·2		10·4		10·1		<0·001
Heavy drinkers (%)	7·3		2·7		1·0		0·4		<0·001
Physically inactive (%)	10·5		10·6		9·7		6·8		0·01
Manual social class (%)	56·4		51·6		47·2		42·0		<0·001
Obesity (BMI≥30 kg/m^2^) (%)	21·2		17·2		15·4		10·8		<0·001
SBP (mmHg)	151·3	22·8	150·1	23·9	149·6	24·3	149·6	24·6	0·13
HDL (mmol/l)	1·4	0·4	1·3	0·3	1·3	0·3	1·3	0·3	0·004
Glucose (mmol/l)	6·1	1·9	6·0	1·8	5·9	1·6	5·9	1·9	0·04
Diabetes (%)	9·5		6·7		5·4		4·0		<0·001
CRP (mg/l)*									<0·001
Mean	1·8		1·7		1·6		1·5		
IQR	0·9–3·7		0·9–3·4		0·7–3·2		0·7–2·9		
vWF (IU/dl)	135·9	47·2	137·9	42·7	136·0	44·7	136·8	45·8	0·91

SBP, systolic blood pressure; CRP, C-reactive protein; vWF, von Willebrand factor.

* Log transformed.

There were a total of 899 deaths, 316 CVD deaths, 569 CVD events and 301 CHD events during a mean period of 11·3 years of follow-up. A ‘high-fat/low-fibre’ dietary pattern was associated with a graded increase in all-cause mortality risk. Although attenuated slightly, this association remained after adjustment for socio-demographic, behavioural and cardiovascular risk factors, with a 44 % increase in risk in the highest compared with the lowest quartile of adherence to the ‘high-fat/low-fibre’ pattern (HR 1·44; 95 % CI 1·13, 1·84, *P*_trend_=0·007). In age-adjusted analysis, participants in the highest quartile of the ‘high-fat/low-fibre’ dietary pattern had an increased risk of CVD mortality with a significant trend (*P*=0·002) and incident CVD events with a borderline significant trend (*P*=0·06). However, these trends disappeared after adjustment for energy intake, smoking, alcohol, physical activity, social class and BMI. No significant trends were seen between the ‘high-fat/low-fibre’ dietary pattern and risk of incident CHD events (Table 4).

**Table 4:** CHD events, CVD events, CVD mortality and all-cause mortality by quartiles (Q) of a ‘high-fat/low-fibre’ dietary pattern in the British Regional Heart Study participants from baseline (1998–2000) to 2010† (Hazard ratios (HR) and 95 % confidence intervals)

	‘High-fat/low-fibre’		Rate (per 1000	Model 1		Model 2		Model 3		Model 4	
	diet quartiles	Cases (*n*)	person years)	HR	95 % CI	HR	95 % CI	HR	95 % CI	HR	95 % CI
All-cause mortality	Q1	187	22·65	1·00		1·00		1·00		1·00	
	Q2	199	24·62	1·06	0·87, 1·30	1·01	0·82, 1·25	1·07	0·85, 1·33	1·10	0·88, 1·38
	Q3	239	30·59	1·32*	1·09, 1·60	1·16	0·94, 1·43	1·12	0·90, 1·40	1·11	0·88, 1·39
	Q4	274	35·69	1·63*	1·36, 1·97	1·40*	1·11, 1·76	1·43*	1·12, 1·82	1·44*	1·13, 1·84
	*P*_trend_			<0·001		0·002		0·005		0·007	
CVD mortality	Q1	62	7·51	1·00		1·00		1·00		1·00	
	Q2	80	9·90	1·27	0·91, 1·78	1·28	0·89, 1·83	1·38	0·94, 2·01	1·45	0·99, 2·12
	Q3	81	10·37	1·32	0·95, 1·84	1·11	0·76, 1·61	1·10	0·74, 1·63	1·10	0·74, 1·64
	Q4	93	12·11	1·67*	1·21, 2·30	1·40	0·94, 2·10	1·36	0·89, 2·09	1·39	0·90, 2·14
	*P*_trend_			0·002		0·19		0·34		0·35	
CVD events	Q1	133	16·95	1·00		1·00		1·00		1·00	
	Q2	139	17·84	1·02	0·81, 1·30	1·00	0·78, 1·30	1·00	0·76, 1·30	1·02	0·78, 1·33
	Q3	146	19·51	1·12	0·88, 1·42	1·01	0·77, 1·31	0·95	0·72, 1·25	0·95	0·72, 1·25
	Q4	151	20·55	1·23	0·97, 1·55	1·04	0·78, 1·40	0·95	0·70, 1·29	0·95	0·69, 1·30
	*P*_trend_			0·06		0·80		0·69		0·64	
CHD events	Q1	70	8·66	1·00		1·00		1·00		1·00	
	Q2	72	9·05	1·02	0·73, 1·41	1·00	0·70, 1·42	1·00	0·69, 1·44	1·02	0·71, 1·48
	Q3	77	10·04	1·13	0·82, 1·56	1·01	0·70, 1·44	0·94	0·64, 1·36	0·92	0·63, 1·35
	Q4	82	10·97	1·28	0·93, 1·76	1·09	0·73, 1·62	0·97	0·63, 1·47	0·95	0·62, 1·46
	*P*_trend_			0·10		0·69		0·80		0·72	

*P<0·05.

† Model 1: age adjusted; model 2: adjusted for model 1+energy intake, smoking status, alcohol intake, physical activity, social class and BMI; model 3: adjusted for model 2+HDL, systolic blood pressure and diabetes; model 4: adjusted for model 3+C-reactive protein and von Willebrand factor.

Adherence to the ‘prudent’ dietary pattern was associated with a significant graded decrease in risk of all-cause mortality across quartiles (*P*_trend_=0·001). However, after adjustment for socio-demographic, behavioural and cardiovascular risk factors (model 4), HR was attenuated slightly, but men in the second quartile of the ‘prudent’ diet still had a significantly decreased risk of all-cause mortality (second *v.* first quartile; HR 0·77; 95 % CI 0·63, 0·95), although the trend across quartiles was not significant (*P*_trend_=0·28). Similar associations were observed with CVD mortality; in the fully adjusted model, men in the second quartile of adherence to a ‘prudent’ diet had a lower risk but the trend across quartiles was not significant (second *v.* first quartile; HR 0·68; 95 % CI 0·47, 0·98, *P*_trend_=0·74). No significant associations were seen between quartiles of a ‘prudent’ diet and the risk of either incident CVD events or incident CHD events (Table 5).

**Table 5:** CHD events, CVD events, CVD mortality and all-cause mortality by quartiles (Q) of a ‘prudent’ dietary pattern in the British Regional Heart Study participants from baseline (1998–2000) to 2010† (Hazard ratios (HR) and 95 % confidence intervals)

	‘Prudent’ diet		Rate (per 1000	Model 1		Model 2		Model 3		Model 4	
	quartiles	Cases (*n*)	person years)	HR	95 % CI	HR	95 % CI	HR	95 % CI	HR	95 % CI
All-cause mortality	Q1	280	36·66	1·00		1·00		1·00		1·00	
	Q2	214	26·64	0·72*	0·60, 0·86	0·76*	0·63, 0·93	0·76*	0·62, 0·93	0·77*	0·63, 0·95
	Q3	202	25·15	0·76*	0·63, 0·91	0·88	0·72, 1·07	0·89	0·72, 1·10	0·93	0·75, 1·14
	Q4	203	24·97	0·72*	0·60, 0·86	0·81*	0·66, 1·00	0·78*	0·62, 0·98	0·83	0·66, 1·04
	*P*_trend_			0·001		0·13		0·11		0·28	
CVD mortality	Q1	95	12·44	1·00		1·00		1·00		1·00	
	Q2	66	8·22	0·66*	0·48, 0·90	0·68*	0·48, 0·95	0·66*	0·46, 0·94	0·68*	0·47, 0·98
	Q3	78	9·71	0·91	0·67, 1·23	0·99	0·71, 1·37	0·98	0·69, 1·39	1·03	0·72, 1·47
	Q4	77	9·47	0·81	0·60, 1·10	0·92	0·64, 1·30	0·87	0·60, 1·27	0·94	0·64, 1·37
	*P*_trend_			0·47		0·89		0·96		0·74	
CVD events	Q1	158	21·64	1·00		1·00		1·00		1·00	
	Q2	140	18·39	0·85	0·68, 1·07	0·92	0·72, 1·17	0·90	0·70, 1·16	0·93	0·72, 1·21
	Q3	133	17·21	0·87	0·69, 1·10	0·92	0·71, 1·18	0·91	0·70, 1·19	0·93	0·71, 1·21
	Q4	138	17·63	0·85	0·68, 1·07	0·93	0·72, 1·22	0·90	0·68, 1·19	0·94	0·71, 1·25
	*P*_trend_			0·22		0·63		0·50		0·68	
CHD events	Q1	87	11·65	1·00		1·00		1·00		1·00	
	Q2	79	10·12	0·87	0·64, 1·18	0·97	0·70, 1·34	0·95	0·68, 1·34	0·99	0·70, 1·40
	Q3	70	8·87	0·83	0·61, 1·14	0·90	0·64, 1·27	0·89	0·61, 1·28	0·92	0·64, 1·34
	Q4	65	8·10	0·73	0·53, 1·01	0·82	0·56, 1·18	0·81	0·55, 1·20	0·86	0·58, 1·27
	*P*_trend_			0·06		0·26		0·27		0·40	

*P<0·05.

† Model 1: age adjusted; model 2: adjusted for model 1+energy intake, smoking status, alcohol intake, physical activity, social class and BMI; model 3: adjusted for model 2+HDL, systolic blood pressure and diabetes; model 4: adjusted for model 3+C-reactive protein and von Willebrand factor.

Adherence to a ‘high-sugar’ dietary pattern was not significantly associated with all-cause mortality (highest *v.* lowest quartile; HR 1·00; 95 % CI 0·77, 1·29, *P*_trend_=0·71) in models adjusted for cardiovascular risk factors (model 4). Although the risk of CVD mortality was increased in men in the top quartile of adherence to the ‘high-sugar’ pattern, there was no significant trend across quartiles (highest *v.* lowest quartile; HR 1·32; 95 % CI 0·84, 2·05, *P*_trend_=0·33). However, a borderline significant trend was observed between adherence to a ‘high-sugar’ diet and an increased risk of both incident CVD events (highest *v.* lowest quartile; HR 1·47; 95 % CI 1·06, 2·04, *P*_trend_=0·06) and incident CHD events (highest *v.* lowest quartile; HR 1·57; 95 % CI 1·00, 2·46, *P*_trend_=0·06) in fully adjusted models (Table 6).

**Table 6:** CHD events, CVD events, CVD mortality and all-cause mortality by quartiles (Q) of a ‘high-sugar’ dietary pattern in the British Regional Heart Study participants from baseline (1998–2000) to 2010† (Hazard ratios (HR) and 95 % confidence intervals)

	‘High-sugar’		Rate (per 1000	Model 1		Model 2		Model 3		Model 4	
	diet quartiles	Cases (*n*)	person years)	HR	95 % CI	HR	95 % CI	HR	95 % CI	HR	95 % CI
All-cause mortality	Q1	219	27·32	1·00		1·00		1·00		1·00	
	Q2	222	28·00	0·94	0·78, 1·14	1·00	0·82, 1·23	1·04	0·84, 1·29	1·06	0·85, 1·31
	Q3	214	26·56	0·81*	0·67, 0·98	0·85	0·68, 1·05	0·89	0·71, 1·12	0·91	0·72, 1·15
	Q4	244	31·18	0·88	0·73, 1·05	0·89	0·70, 1·13	0·96	0·75, 1·24	1·00	0·77, 1·29
	*P*_trend_			0·08		0·18		0·53		0·71	
CVD mortality	Q1	64	7·98	1·00		1·00		1·00		1·00	
	Q2	73	9·21	1·04	0·74, 1·45	1·07	0·75, 1·53	1·10	0·75, 1·63	1·13	0·76, 1·66
	Q3	75	9·31	0·91	0·66, 1·29	0·91	0·62, 1·33	0·95	0·63, 1·43	0·97	0·64, 1·47
	Q4	104	13·29	1·18	0·86, 1·61	1·07	0·71, 1·61	1·27	0·82, 1·96	1·32	0·84, 2·05
	*P*_trend_			0·39		0·94		0·40		0·33	
CVD events	Q1	112	14·50	1·00		1·00		1·00		1·00	
	Q2	139	18·34	1·17	0·91, 1·50	1·26	0·97, 1·65	1·22	0·92, 1·62	1·27	0·95, 1·68
	Q3	139	18·00	1·07	0·83, 1·37	1·10	0·83, 1·46	1·08	0·80, 1·46	1·10	0·81, 1·50
	Q4	179	24·04	1·32*	1·04, 1·67	1·33	0·98, 1·81	1·40*	1·01, 1·93	1·47*	1·06, 2·04
	*P*_trend_			0·05		0·16		0·09		0·06	
CHD events	Q1	59	7·48	1·00		1·00		1·00		1·00	
	Q2	67	8·61	1·08	0·76, 1·53	1·11	0·76, 1·61	1·13	0·76, 1·68	1·15	0·77, 1·72
	Q3	79	10·00	1·18	0·84, 1·65	1·24	0·85, 1·82	1·21	0·80, 1·83	1·20	0·79, 1·83
	Q4	96	12·60	1·40*	1·01, 1·94	1·42	0·93, 2·16	1·52	0·97, 2·38	1·57	1·00, 2·46
	*P*_trend_			0·04		0·09		0·07		0·06	

*P<0·05.

† Model 1: age adjusted; model 2: adjusted for model 1+energy intake, smoking status, alcohol intake, physical activity, social class and BMI; model 3: adjusted for model 2+HDL, systolic blood pressure and diabetes; model 4: adjusted for model 3+C-reactive protein and von Willebrand factor.

## Discussion

In this study, principal component analysis was used to apply *a posteriori*-defined dietary patterns to a cohort of older men, identifying three interpretable dietary patterns: ‘high fat/low fibre’, ‘prudent’ and ‘high sugar’. Adherence to a ‘high-fat/low-fibre’ pattern (a high intake of red meat, meat products, white bread, fried potato and eggs) was associated with an increased risk of all-cause mortality, with a 44 % higher risk in those in the highest compared with the lowest quartile of adherence. However, adherence to a ‘prudent’ diet (characterised by a high consumption of poultry, fish, fruits, vegetables, pasta, rice and wholemeal bread) did not show a significant trend with cardiovascular outcomes or mortality in adjusted analysis. The third dietary pattern identified was a ‘high-sugar’ pattern (characterised by a high consumption of biscuits, puddings, chocolate, sweets), which was associated with a borderline significant trend for an increased risk of CVD events and CHD events in fully adjusted models. This study adds to the limited literature describing *a posteriori* dietary patterns and assessing associations with the risk of CVD and mortality in older British adults.

The dietary patterns that emerged in this study are consistent with those reported in previous studies using principal component analysis, which have typically identified two major types of dietary patterns, an unhealthy/Western diet and a healthy/prudent diet^(^ ^13^ ^–^ ^15^ ^)^. The Hertfordshire Cohort Study, comprising older British adults aged 59–73 years, identified very similar dietary patterns (prudent and traditional)^(^ ^37^ ^)^ to those identified in this study. In this previous study, the prudent diet (high in consumption of fruit, vegetables, oily fish and wholemeal cereals) is comparable with the ‘prudent’ diet observed in this cohort and adherence was also associated with non-manual social class and being a non-smoker. The traditional diet (high in consumption of vegetables, processed and red meat, fish and puddings) had similarities with the ‘high-fat/low-fibre’ diet in this cohort and was also associated with higher alcohol consumption. The three dietary patterns identified in this study together explained about 21 % of the total variance in the dietary data. Although this proportion of variance seems low, this is actually greater than the variance explained by a comparable study in older British adults, aged 65 years and over, which identified four interpretable principal components from the National Diet and Nutrition Survey data, explaining 9·8 % of the total variance^(^ ^16^ ^)^.

Results of this study showed a significant graded association between adherence to a ‘high-fat/low-fibre’ dietary pattern and an increased risk of all-cause mortality. These results are consistent with studies showing that fibre intake and all-cause mortality are inversely associated^(^ ^38^ ^)^, and that the consumption of red meat and processed meat (two components of the ‘high-fat/low-fibre’ dietary patterns with high factor loadings) is associated with an increased risk of all-cause and cancer mortality^(^ ^39^ ^,^ ^40^ ^)^. Adherence to a ‘prudent’ diet was associated with a non-significantly reduced risk of all-cause mortality; there was also no significant trend between a ‘high-sugar’ diet and the risk of all-cause mortality. None of the three dietary patterns described here were associated with CVD mortality. A comparable study of an older British population, aged 65 years and older, from the National Diet and Nutrition Survey, identified four interpretable diet patterns using principal component analysis: ‘Mediterranean style’, ‘health aware’, ‘traditional’ and ‘sweet and fat’. Only the Mediterranean-style dietary pattern was associated with a reduced risk of all-cause mortality, with an 18 % reduction in risk in those in the highest compared with the lowest tertile^(^ ^16^ ^)^. However, in men only, the traditional diet was also a risk factor for mortality, similar to the ‘high-fat/low-fibre’ results observed in this cohort.

A recent meta-analysis of thirteen prospective studies involving 338 787 participants examined the association between dietary patterns defined by principal component analysis and risk of all-cause and CVD mortality^(^ ^14^ ^)^. Summary relative risk estimates (SRRE) showed a significant inverse association between a prudent/healthy dietary pattern and all-cause mortality (highest *v.* lowest category of dietary pattern score; SRRE 0·76; 95 % CI 0·68, 0·86) and CVD mortality (SRRE 0·81; 95 % CI 0·75, 0·87) but non-significant associations between the Western/unhealthy dietary pattern and all-cause mortality (SRRE 1·07; 95 % CI 0·96, 1·20) and CVD mortality (SRRE 0·99; 95 % CI 0·91, 1·08). The risk estimates observed in the present study for the association between a prudent diet and a lower risk of all-cause mortality and CVD mortality were in the same direction, and of similar magnitudes, as those in the meta-analysis, but statistically non-significant. In addition, a significant association was observed in the present study between higher adherence to an unhealthy diet (the ‘high-fat/low-fibre’ diet) and a higher risk of all-cause mortality, which was not seen in the meta-analysis. These discrepancies may have been due to the much bigger sample size of the meta-analysis giving greater power to detect smaller effects or due to the differences in confounder adjustments; few studies included in this meta-analysis had such a comprehensive adjustment for established and emerging cardiovascular risk factors such as those included in this study.

This study found no significant association between a ‘high-fat/low-fibre’ diet or a ‘prudent’ diet and the risk of CVD events or CHD events, but adherence to a ‘high-sugar’ diet was associated with a borderline significant trend for an increased risk of CVD events and CHD events in fully adjusted models. These results are in keeping with the American Heart Association recommendation of reducing dietary intake of added sugars in order to lower the risk of CVD^(^ ^41^ ^)^ and the recent suggestion that sugar may be a more important risk factor than fat for CVD^(^ ^42^ ^,^ ^43^ ^)^. A meta-analysis of twelve prospective studies involving 409 780 participants examined the association between principal component analysis-defined dietary patterns and CHD risk. Summary relative risks showed an inverse association between the prudent/healthy diet and CHD risk, but no association with the Western/unhealthy diet^(^ ^15^ ^)^. The observed association between a healthy diet and CHD events in this meta-analysis, but not in the current study, may again possibly be explained by the much larger sample size in the meta-analysis giving greater power to detect smaller effects. This meta-analysis did not identify any studies from the UK specifically and did not mention high-sugar/sweet dietary patterns, as observed in this cohort. Results from this study may therefore be the first study of principal component analysis-defined dietary patterns and CHD risk in the UK, and this study has shown that a high-sugar dietary pattern may increase CHD and CVD risk in older British men. These analyses should therefore be replicated in other older adult cohorts.

A major strength of this study is that data are from a moderately large prospective population-based study, with negligible loss to follow-up and objective ascertainment of CVD and mortality outcomes^(^ ^20^ ^,^ ^36^ ^)^. However, the study comprised predominately white European older male participants, and therefore the applicability of findings to women and non-white ethnic groups is uncertain. Dietary intake was assessed using a FFQ, which has previously been validated against weighed food intakes in British populations^(^ ^22^ ^,^ ^23^ ^)^, and the dietary intake of participants was broadly comparable with those from the National Diet and Nutrition Survey^(^ ^44^ ^)^. However, FFQ are more prone to measurement error compared with some other dietary measures, and in older populations non-response to FFQ could have increased the chance of dietary under-reporting^(^ ^45^ ^,^ ^46^ ^)^. However, no significant difference was seen in participants with and without complete food group data in terms of diet quality, assessed by daily fruit and vegetable intake, or the risk of CVD events. Another consideration in this study is that the FFQ measured dietary intake at baseline only, and therefore whether dietary patterns of participants changed throughout follow-up was unknown. It is possible that reverse causation may have existed in this study – for example, diabetics may have changed their dietary patterns following diagnosis, and this may explain the unexpected inverse association observed between prevalent diabetes at baseline and the high-fat/low-fibre dietary pattern. However, such effects are only likely to have biased prospective associations between dietary patterns and outcomes towards the null.

*A posteriori* methods of defining dietary patterns have an advantage over using *a priori* methods, of making no previous assumptions about dietary patterns, instead using an empirical, data-driven approach to derive typical patterns of dietary intake^(^ ^9^ ^)^. However, data on the reproducibility and validity of the principal component method are limited^(^ ^47^ ^–^ ^49^ ^)^, and subjectivity was introduced at various points in the analysis, such as the grouping of dietary variables and the choice of how many components to retain, which may have influenced the observed associations with disease risk^(^ ^9^ ^,^ ^29^ ^,^ ^30^ ^)^. However, the food groups used here were comparable with those used previously for a nationally representative dietary survey of British adults^(^ ^27^ ^)^, and criteria for deciding how many components to retain were decided in advance of the analysis, both of which helped to reduce this bias.

Dietary patterns persist as an important risk factor for CVD and all-cause mortality in the elderly, with higher adherence to a ‘high-fat/low-fibre’ dietary pattern being associated with an increased risk of all-cause mortality, and higher adherence to a ‘high-sugar’ diet being associated with a modest increase in risk of CVD events and CHD events, which could not be explained by adjustment for cardiovascular risk factors. The ‘prudent’ diet was not significantly associated with cardiovascular outcomes or mortality. Adopting a diet that avoids ‘high-fat/low-fibre’ and ‘high-sugar’ components may reduce the risk of cardiovascular events and all-cause mortality in older adults.

## Supplementary material

For supplementary material accompanying this paper visit http://dx.doi.org/10.1017/S0007114516003147. click here to view supplementary material
