# Supplementary material for: Dietary patterns and the risk of CVD and all-cause mortality in older British men
Source: Br J Nutr. 2016 Sep 13;116(7):1246–55. doi: 10.1017/S0007114516003147 (PMC5053073; doi:10.1017/S0007114516003147)
Supplement: Supplementary file 1 [file S0007114516003147sup001.docx]

###### ONLINE SUPPLEMENTARY MATERIAL

###### SUPPLEMENTAL TABLE 1 List of 34 food groups derived from items in the food frequency questionnaire, used in British Regional Heart Study participants in1998-2000

| **Food group** | **Food items** | **Units*** | **Range** |
| --- | --- | --- | --- |
| Red meat | Beef (including minced beef, beef burgers); lamb; pork, bacon, ham, salami | days/week | 0-7 |
| Poultry | Chicken, turkey, other poultry | days/week | 0-7 |
| Meat products | Tinned meat (all types, corned beef, etc.); pork sausages; beef sausages; meat pies, pasties; liver, kidney, heart | days/week | 0-7 |
| Fish | White fish (cod, haddock, hake, plaice, fish fingers, etc.); kippers, herrings, pilchards, tuna, sardines, salmon, mackerel (including tinned); shellfish | days/week | 0-7 |
| Potatoes | Boiled, baked, mashed | days/week | 0-7 |
| Fried potatoes | Chips or fried (from shop); chips or fried (cooked at home); roast potatoes | days/week | 0-7 |
| Vegetables | Green vegetables, salad; carrots; parsnips, swedes, turnips, beetroot, other root vegetables; onions; tomatoes | days/week | 0-7 |
| Legumes | Baked or butter beans, lentils, peas, chickpeas, sweetcorn | days/week | 0-7 |
| Fruit | Apples; pears; oranges; bananas; other fruits | pieces/week | 0-60 |
| Pasta and rice | Spaghetti and other pasta; rice (all types except pudding rice) | days/week | 0-7 |
| Breakfast cereal | Grapenuts, porridge Ready Brek, Special K, Sugar Puffs, Rice Crispies; Cornflakes, muesli, Shredded Wheat, Sultana Bran, Weetabix; Bran Flakes, Puffed Wheat; All Bran, Wheat Bran; other cereal | days/week | 0-7 |
| White bread | White bread | days/week | 0-7 |
| Wholemeal bread | Brown bread; wholemeal bread | days/week | 0-7 |
| Full-fat cheese | e.g. cheddar, Leicester, stilton, brie, soft cheeses | days/week | 0-7 |
| Low-fat cheese | e.g. edam, cottage cheese, reduced fat cheeses | days/week | 0-7 |
| Full-fat milk | Full-fat milk | None; ≤0.5 pint; 0.5-1 pint; >1 pint/day  None; ≤0.5 pint; 0.5-1 pint; >1 pint/day  None; ≤0.5 pint; 0.5-1 pint; >1 pint/day  None; ≤0.5 pint; 0.5-1 pint; >1 pint/day | 1-4 |
| Semi-skimmed milk | Semi-skimmed milk |  | 1-4 |
| Skimmed milk | Skimmed milk |  | 1-4 |
| Other milk | Condensed milk, evaporated milk etc |  | 1-4 |
| Biscuits and puddings | Digestive biscuits, plain biscuits; sweet biscuits, sponge cakes, scones, buns; ice-cream, sweet yoghurts, trifle; fruit cake, fruit bread, plum pudding; fruit tart, jam tart, fruit crumble; milk puddings (rice, tapioca); tinned fruit, jellies; sweet sauces (chocolate, custard) | days/week | 0-7 |
| Chocolate and sweets | Chocolate, chocolate bars, sweets (all types) | days/week | 0-7 |
| Eggs | Eggs (boiled, poached, fried, scrambled); eggs in baked dishes (e.g. flans, quiches, soufflés, egg custard etc.) | days/week | 0-7 |
| Fruit juice | Natural fruit juices (including tomato juice) | days/week | 0-7 |
| Soft drinks | Fizzy drinks, non-diet squashes; low calorie (diet) squashes and fizzy drinks | days/week | 0-7 |
| Tea and coffee | Tea; coffee; other hot drinks (hot chocolate, malted milk, Horlicks) | cups/day | 0-34 |
| Nuts | Nuts (e.g. salted or unsalted peanuts), nut butter | days/week | 0-7 |
| Savoury snacks | Potato crisps, corn chips, crackers | days/week | 0-7 |
| Sweet spreads | Jam, honey, marmalade, chocolate spread | days/week | 0-7 |
| Sauces and soups | Chutney, brown sauce, tomato sauce; soups (all kinds, home-made, tinned, packet) | days/week | 0-7 |
| Wine | Wine | single glasses/week | 0-44 |
| Beer | Beer, Lager, Shandy | pints/week | 0-70 |
| Spirits | Spirits | single glasses/week | 0-84 |
| Olive oil | Olive oil | mls/week | 0-500 |
| Butter | Butter | grams/week | 0-1134 |

*Units of measurements based on the available frequency measures from the food frequency questionnaire.

###### SUPPLEMENTAL TABLE 2 Food group factor loadings for ‘high fat/low fibre’, ‘prudent’ and ‘high sugar’ dietary patterns, in British Regional Heart Study participants in 1998-2000^a^

| **Food groups** | **‘High fat/low fibre’ dietary pattern** | **‘Prudent’ dietary pattern** | **‘High sugar’ dietary pattern** |
| --- | --- | --- | --- |
|  |  |  |  |
| Variance explained (%) | 7.9 | 7.1 | 5.8 |
|  |  |  |  |
| Red meat | **0.33** | 0.12 | 0.03 |
| Poultry | 0.03 | **0.29** | -0.09 |
| Meat products | **0.42** | 0.08 | -0.04 |
| Fish | 0.07 | **0.38** | -0.08 |
| Potatoes | 0.05 | 0.12 | 0.12 |
| Fried potatoes | **0.36** | 0.01 | 0.02 |
| Vegetables | -0.01 | **0.26** | 0.09 |
| Legumes | 0.14 | **0.26** | 0.01 |
| Fruit | -0.13 | **0.23** | 0.04 |
| Pasta and rice | 0.00 | **0.34** | -0.06 |
| Breakfast cereal | -0.19 | 0.06 | **0.28** |
| White bread | **0.34** | -0.19 | 0.04 |
| Wholemeal bread | **-0.30** | **0.25** | 0.07 |
| Full-fat cheese | 0.04 | 0.05 | **0.25** |
| Low-fat cheese | -0.03 | 0.17 | -0.03 |
| Full-fat milk | 0.04 | -0.07 | 0.13 |
| Semi-skimmed milk | 0.03 | -0.01 | 0.13 |
| Skimmed milk | -0.13 | 0.10 | -0.17 |
| Other milk | 0.07 | 0.03 | -0.12 |
| Biscuits and puddings | 0.06 | -0.03 | **0.46** |
| Chocolate and sweets | 0.06 | -0.04 | **0.41** |
| Eggs | **0.26** | **0.23** | 0.09 |
| Fruit juice | -0.13 | 0.17 | 0.12 |
| Soft drinks | 0.08 | 0.05 | 0.09 |
| Tea and coffee | 0.09 | -0.06 | 0.12 |
| Nuts | 0.01 | 0.14 | 0.10 |
| Savoury snacks | 0.16 | 0.03 | 0.15 |
| Sweet spreads | -0.12 | 0.06 | **0.36** |
| Sauces and soups | 0.17 | **0.26** | 0.18 |
| Wine | -0.18 | 0.19 | -0.04 |
| Beer | **0.23** | 0.09 | **-0.31** |
| Spirits | 0.01 | 0.10 | -0.11 |
| Olive oil | -0.08 | **0.23** | -0.05 |
| Butter | 0.09 | 0.02 | 0.04 |

^a^Factor loadings ≥ 0.20 / ≤ -0.20 are indicated in bold.
